# Identification of the interplay between SOX9 and S100P in the metastasis and invasion of colon carcinoma

**DOI:** 10.18632/oncotarget.3967

**Published:** 2015-05-13

**Authors:** Zhiyong Shen, Haijun Deng, Yuan Fang, Xianjun Zhu, Geng-tai Ye, Li Yan, Hao Liu, Guoxin Li

**Affiliations:** ^1^ Department of General Surgery, Nanfang Hospital, Southern Medical University, Guangzhou, China; ^2^ Department of Gastroenterology, Nanfang Hospital, Southern Medical University, Guangzhou, China

**Keywords:** SOX9, S100P, transcriptional regulation, metastasis and invasion, epithelial-mesenchymal transition

## Abstract

Elevated expression of S100P has been detected in several tumor types and suggested to be responsible for tumor metastasis and invasion, but the upstream regulatory mechanisms promoting S100P overexpression are largely unknown. Here, we report that SOX9 was predicted and verified as a transcription factor of S100P. SOX9 and S100P were both overexpressed in colon cancer. SOX9 bound to and activated the S100P promoter. Knockdown of SOX9 expression down-regulated S100P expression, resulting in reduced invasiveness and metastasis of colon cancer cells by inhibiting the activation of receptor for advanced glycation end products (RAGE)/ERK signaling and epithelial-mesenchymal transition (EMT). Further, decreased expression of SOX9 dramatically inhibited the tumor growth and peritoneal metastasis in nude mice. More importantly, S100P was found to be critical for SOX9-mediated metastasis and invasion in colon cancer. Knockdown of S100P in SOX9-overexpressing colon cancer cells dramatically suppressed metastasis and invasion both *in vitro* and in mice. We also detected SOX9 and S100P expression in a tissue microarray with 90 colon cancer cases to provide their clinical relevance. There was a strong correlation between SOX9 and S100P expression in colon carcinomas. In conclusion, our results suggest that SOX9 promotes tumor metastasis and invasion through regulation of S100P expression.

## INTRODUCTION

S100P is a calcium-binding protein that belongs to the S100 protein family, which is characterized by two Helix-E and Helix-F loop-hand calcium-binding motifs that mediate Ca^2+^-dependent signal transduction [1]. Most studies have indicated that overexpression of S100P correlates with proliferation, tumorigenesis regulation, invasion, metastasis, and cancer cell motility. We previously investigated differences in the gene expression profiles of colon cancer and normal tissues using gene microarray analysis, followed by confirmation by real-time quantitative polymerase chain reaction (Q-PCR). Q-PCR analysis revealed an average 3.5-fold overexpression of S100P in colon cancer.

To investigate the regulation of the S100P overexpression, we searched for transcription factors of the S100P gene. Bioinformatic analysis and literature searches identified SOX9 as a potential transcription factor. SOX9, a high mobility group (HMG) box transcription factor, is highly up-regulated in many premalignant lesions and in tumor tissues and has been proposed to play an oncogenic role in tumor development [2, 3]. The co-expression of exogenous SOX9 and Slug transforms differentiated luminal cells into mammary stem cells (MaSCs), promotes the tumorigenic and metastasis-seeding abilities of human breast cancer cells, and is associated with poor patient survival [4]. SOX9 is also correlated with Wnt/b-catenin activation, which induces increased LRP6 and TCF4 expression in breast cancer [5]. A relationship between SOX9 and colon cancer growth and progression as well as colonic epithelial stem cell differentiation has also been reported [3, 6, 7]. Together, these findings led us to hypothesize that SOX9 may directly or indirectly regulate S100P and may be associated with aggressive phenotypes of colon cancer.

In line with this hypothesis, we demonstrated that overexpression of SOX9 induces the expression of S100P in both a colon cancer cell line and human samples, leading to increased cell invasiveness and metastasis as well as activation of epithelial-mesenchymal transition (EMT). Expression of SOX9 and S100P is strongly correlated in primary colon cancer tissues and is associated with adverse prognosis.

## RESULTS

### SOX9 binds to the S100P promoter and up-regulates its transcription activity

**Figure 1:**
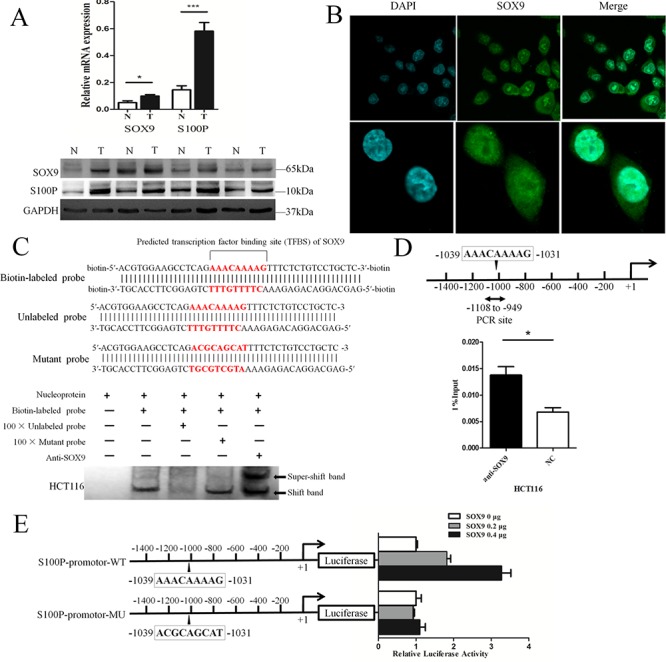
SOX9 binds directly to the *S100P* promoter and up-regulates its transcriptional activity **A.** SOX9 and S100P mRNA and protein were both overexpressed in colon carcinoma as detected by Q-PCR and Western blotting, respectively. N: normal tissue, T: tumor. *P < 0.05 and ***P < 0.001. **B.** SOX9 localization in the HCT116 cell line was determined by immunofluorescence. **C.** Biotin-labeled, unlabeled, and mutant probes used for EMSAs were designed based on the predicted transcription factor-binding site (TFBS) of SOX9 in the *S100P* gene promoter (upper). SOX9 formed specific complexes with the biotin-labeled probe as verified by EMSA (lower). The shifted band represents the SOX9/biotin-labeled probe complexes, and the supershifted band represents the SOX9 antibody/SOX9/biotin-labeled probe complexes (arrow). **D.** ChIP assay for SOX9 binding to the S100P promoter. Diagram representing the position of primers used for PCR in the S100P promoter region (upper). ChIP was performed with either anti-SOX9 antibody or control IgG, and Q-PCR analysis of the relative binding affinity of SOX9 to S100P promoter region (lower). NC: negative control. *P < 0.05. **E.** Luciferase activity of wild type and mutant S100P promoter constructs. Schematic diagram showing the potential recognition site (AAACAAAAG) of SOX9 in the wild type S100P promoter and the mutant S100P promoter sequence (left). The luciferase activity of two promoter constructs was measured when co-transfected with increasing amounts of SOX9-overexpressing plasmid (right).

To explore the expression patterns of SOX9 and S100P in colon cancer, we measured the expression levels of SOX9 and S100P in colon cancer tissues by Q-PCR and Western blot analyses. Overexpression of SOX9 (*P* = 0.038) and S100P (*P* < 0.001) mRNA was observed in colon cancer. Western blot analysis showed that SOX9 and S100P proteins were both up-regulated in colon cancer (Figure 1A). The localization of SOX9 in the HCT116 cell line was investigated by immunofluorescence. The results indicated that SOX9 was expressed in the nucleus, which is essential for the proper functioning of transcription factors (Figure 1B).

We identified potential SOX9-binding sites in the upstream sequence of the S100P promoter within the proximal 1.5 kb of the S100P 5′ flanking region (relative to the transcription start site). To identify if SOX9 binds to the S100P promoter *in vitro* and *in vivo*, we performed an electrophoretic mobility shift assay (EMSA) by designing biotin-labeled, unlabeled, and mutant probes based on the predicted transcription factor-binding site (TFBS) of SOX9 for the S100P promoter (Figure 1C, upper). EMSAs revealed that the shifted band, which represents binding of the biotin-labeled probe to SOX9 (Figure 1C, lane 2), showed lower intensity when a 100-fold excess of unlabeled probe was used to compete with the biotin-labeled probe for binding to SOX9 (Figure 1C, lane 3). However, 100-fold excess of the mutant probe, which could not bind to SOX9, did not compete with the biotin-labeled probe (Figure 1C, lane 4). The formation of a supershifted band following the addition of a SOX9 antibody further suggested that the SOX9 protein bound to the biotin-labeled probe (Figure 1C, lane 5). A chromatin immunoprecipitation (ChIP) assay was then performed to verify the interaction between SOX9 and the *S100P* promoter. Cell lysates were sonicated to generate approximately 400 bp chromatin fragments prior to immunoprecipitation. Chromatin that co-immunoprecipitated with anti-SOX9 or a control IgG antibody was amplified by PCR using primers flanking (−1108 to −949 bp, Supplementary Table S1) the putative SOX9-binding site-containing regions (Figure 1D, upper). The ChIP results showed that anti-SOX9 pulled down the protein/*S100P* gene complex from HCT116 cells compared with NC (*P* = 0.018, Figure 1D, lower).

We then assessed the effect of SOX9 overexpression on the activity of the *S100P* promoter. For these studies, reporter genes containing wild type (S100P-promoter-WT) and mutant (S100P-promoter-MU) sequences of the *S100P* promoter were generated (Figure 1E, left). These reporter constructs were co-transfected into HCT116 cells with a plasmid overexpressing SOX9 (or empty vector). SOX9 overexpression significantly increased S100P-promoter-WT reporter gene expression, but SOX9 overexpression did not increase the S100P-promoter-MU reporter gene expression (Figure 1E, right). The significant increase in S100P-promoter-WT reporter activity with elevated expression of SOX9 demonstrated that SOX9 up-regulates *S100P* transcription activity by binding to a specific promoter region.

### Decreased expression of SOX9 down-regulates S100P inhibition of metastasis and invasion of colon cancer cells both *in vitro* and *in vivo*

**Figure 2:**
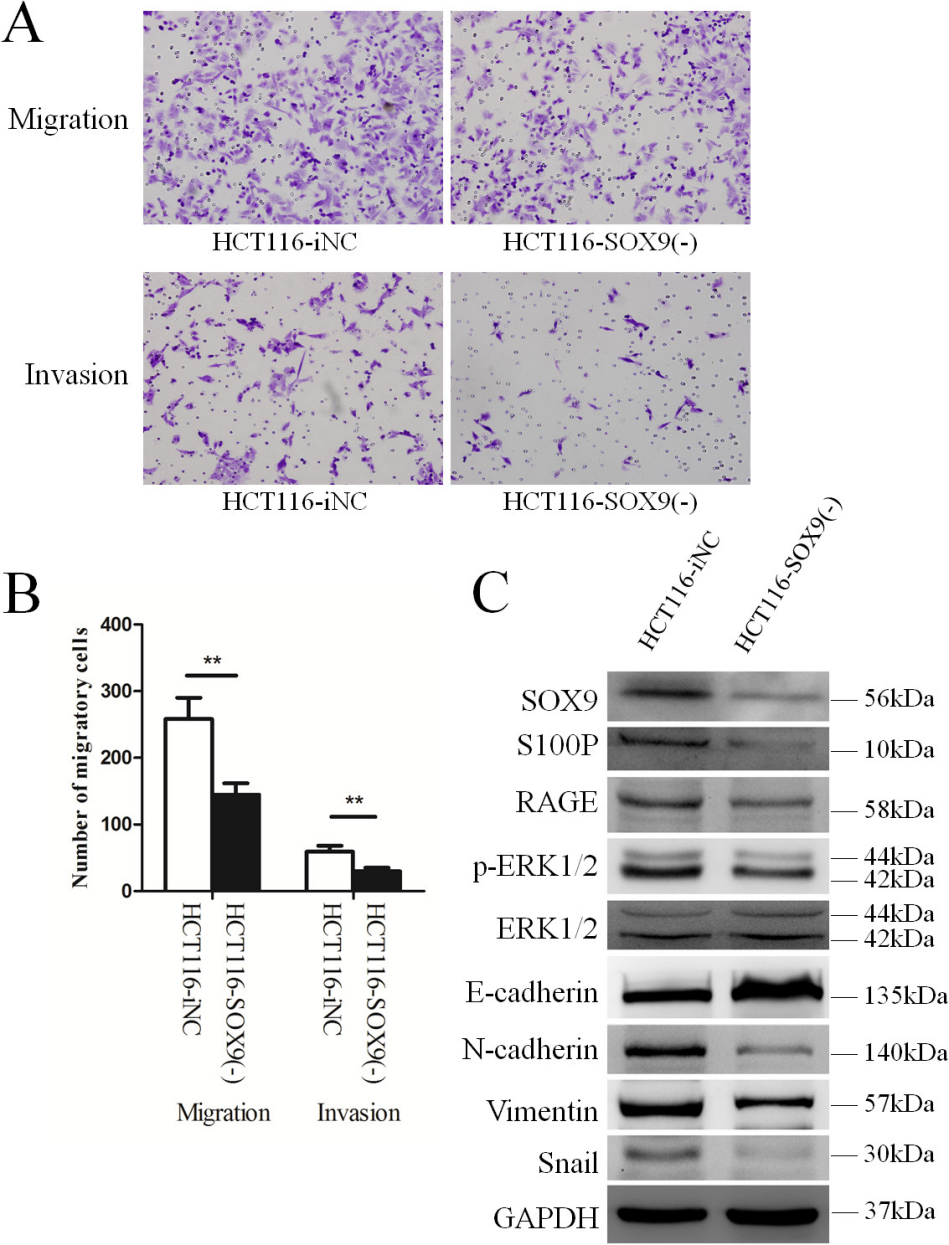
Knockdown SOX9 impairs colon cancer cell migration and invasion via regulation of S100P expression **A.** Effects of SOX9 on cell migration of the HCT116 cell line in Transwell chambers (upper), and its effects on cell invasion in Borden chambers (lower). **B.** The comparison of the average number of cells penetrating the artificial basement membrane in the migration and invasion assays described in A is shown. **P < 0.01. **C.** Decreased expression of SOX9 inhibits the S100P/RAGE/ERK/EMT signaling pathway as detected by Western blot analysis.

The functional effect of SOX9 knockdown on cell behavior in colon cancer cell lines *in vitro* was assessed by transfecting SOX9 siRNA (Supplementary Table S2, Supplementary Figure S1) lentivirus into HCT116 cells to generate stable transfectants (HCT116-SOX9(−)). In the Transwell metastasis assay, HCT116-SOX9(−) cells exhibited lower capacity for migration compared with HCT116-iNC cells (*P* = 0.001, Figure 2A and 2B). In the Matrigel invasion assay, HCT116-SOX9(−) cells exhibited significantly less invasiveness compared with HCT116-iNC cells (*P* = 0.001, Figure 2A and 2B). Western blot analysis revealed that SOX9 and S100P were both down-regulated in HCT116-SOX9(−) cells (Figure 2C). Figure 2C also shows that downstream of S100P, RAGE and phosphorylation levels of ERK1/2 were down-regulated in HCT116-SOX9(−) cells compared with HCT116-iNC cells with no change in the total amount of ERK1/2 protein. Moreover, the expression of a typical EMT epithelial marker, E-cadherin, was up-regulated in HCT116-SOX9(−) cells. In contrast, the mesenchymal markers, N-cadherin, Vimentin, and Snail, were down-regulated upon SOX9 knockdown (Figure 2C).

**Figure 3:**
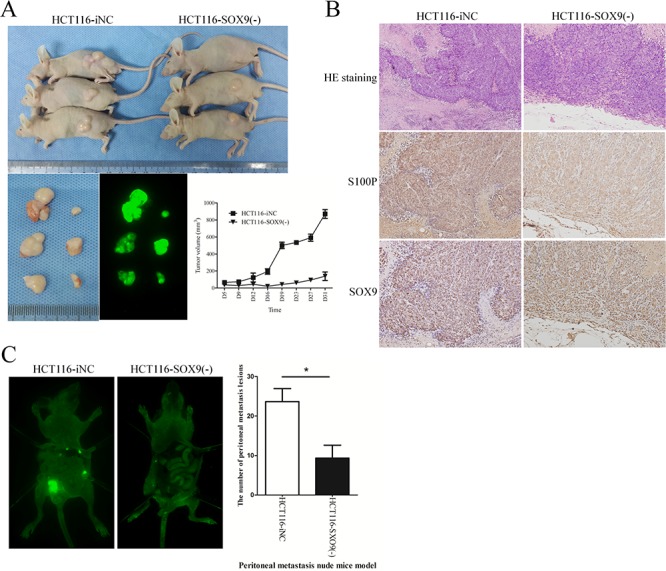
SOX9 enhances tumorigenesis and metastasis *in vivo* **A.** Examination of tumorigenesis in subcutaneously injected animals. Tumor volumes were measured on the indicated days to assess the effects of SOX9 on subcutaneous tumor growth, and fluorescent images of xenograft tumors from NOD-SCID mice subcutaneously injected with HCT116-iNC or HCT116-SOX9(−) cells (4 × 10^6^) were obtained. **B.** HE staining and immunohistochemical staining were performed to assess SOX9 and S100P expression in the subcutaneous tumors of mice injected with HCT116-iNC or HCT116-SOX9(−) cells. **C.** Quantification of the metastatic foci in the abdominal cavity. Whole-body fluorescence images of metastasis in mice intraperitoneally injected with HCT116-iNC or HCT116-SOX9(−) cells (1 × 10^7^) were acquired with the *In-Vivo* F Imaging System (Kodak). The peritoneal metastatic foci were counted and analyzed. *P < 0.05.

To assess the effect of SOX9 on tumorigenesis *in vivo*, HCT116-SOX9(−) and HCT116-iNC cells were implanted subcutaneously into nude mice, and the growth of the resultant primary tumors was monitored. Mice injected with SOX9 knockdown cells developed smaller tumors than those injected with HCT116-iNC cells (*P* < 0.001, Figure 3A). HE and IHC staining verified the positive expression of SOX9 and S100P in the SOX9 knockdown xenografted tumors compared with the negative control (Figure 3B). To determine the effect of SOX9 on the dissemination of metastasis *in vivo*, HCT116-SOX9(−) and HCT116-iNC cells were injected into the abdominal cavities of mice, and the mice were subsequently scanned for metastasis using a whole-body visualization system. Fewer metastatic foci were observed in the abdominal cavities of mice injected with HCT116-SOX9(−) cells compared with those injected with HCT116-iNC cells (*P* = 0.037, Figure 3C). Overall, these results were consistent with our hypothesis that SOX9 induction of S100P overexpression in colon cancer cells stimulates cell migration and invasion through activation of the classical EMT signaling pathway.

### S100P induction is critical for SOX9-mediated cell migration and invasion

**Figure 4:**
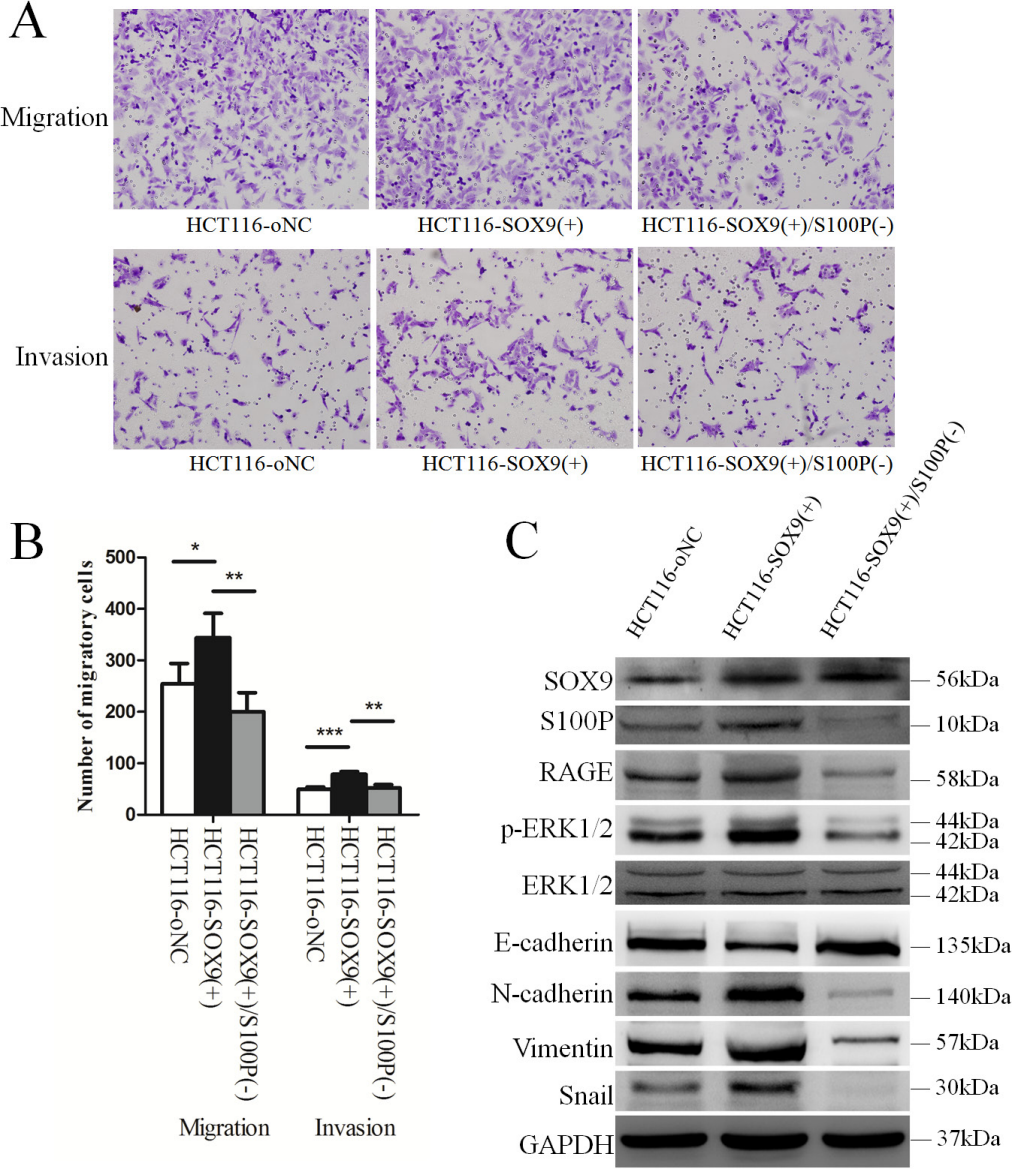
S100P is required for SOX9-induced metastasis and invasion of colon cancer cells **A.** Assessment of the influence of S100P expression on SOX9-induced metastasis and invasion using established stable SOX9 overexpression (HCT116-SOX9(+)) and SOX9 overexpression plus S100P knockdown (HCT116-SOX9(+)/S100P(−)) cell lines by Transwell and Borden chambers. **B.** Comparison of the average number of cells penetrating the artificial basement membrane in the migration and invasion assays described in A. *P < 0.05, **P < 0.01, and ***P < 0.001. **C.** Western blot analysis of the role of S100P in the downstream signaling pathway of SOX9.

Because SOX9 promotes cell migration and invasion, we investigated whether S100P mediates the effect of SOX9 on cell migration and invasion in colon cancer cells. We first constructed a SOX9-overexpressing HCT116 cell line (HCT116-SOX9(+)) and its negative control (HCT116-oNC). We then utilized the lentivirus expressing S100P-specific siRNA to knockdown S100P expression in HCT116-SOX9(+) cells, thus establishing a SOX9-overexpressing and S100P knockdown colon cancer cell line (HCT116-SOX9(+)/S100P(−)). Using these stable cell lines, we performed cell migration and invasion assays, and we found that cell migration (*P* = 0.028) and invasion (*P* < 0.001) of HCT116-SOX9(+) cells were significantly greater than that of HCT116-oNC cells. However, the migration (*P* = 0.003) and invasive (*P* = 0.001) responses were decreased significantly in SOX9-overexpressing cells after S100P expression was suppressed (Figure 4A and 4B). Western blot analysis indicated that SOX9 overexpression promoted EMT by regulating S100P and that S100P knockdown could reverse this phenomenon. The downstream signaling events of S100P and EMT processes were inhibited in HCT116-SOX9(+) cells when S100P was not up-regulated (Figure 4C).

**Figure 5:**
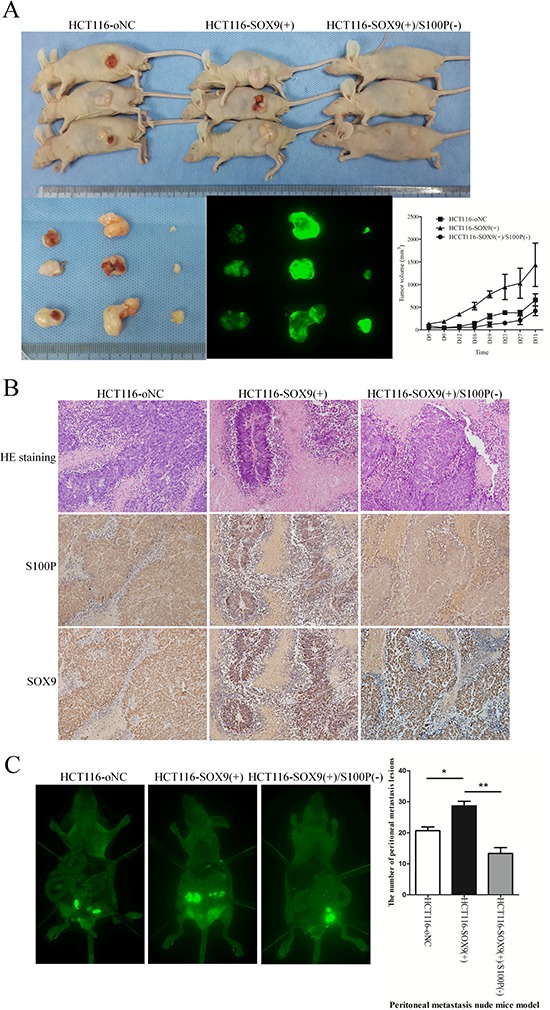
S100P is essential for SOX9 induction of tumorigenesis and metastasis *in vivo* **A.** Evaluation of tumorigenesis in nude mice subcutaneously injected with HCT116-oNC, HCT116-SOX9(+), and HCT116-SOX9(+)/S100P(−) cells (4 × 10^6^). To assess the effects of S100P on subcutaneous tumor growth, tumor volumes were recorded on the indicated days, and fluorescence images of xenograft tumors from NOD-SCID subcutaneously injected were obtained. **B.** Confirmation of SOX9 and S100P expression in the subcutaneous tumors of mice by HE staining and immunohistochemical staining. **C.** Whole-body fluorescence images of metastasis in mice intraperitoneally injected with HCT116-oNC, HCT116-SOX9(+), and HCT116-SOX9(+)/S100P(−) cells (1 × 10^7^) were acquired with the In-Vivo F Imaging System (Kodak). The peritoneal metastatic foci were counted and analyzed. *P < 0.05 and **P < 0.01.

To further investigate whether up-regulation of S100P is necessary for SOX9-induced tumorigenesis and metastasis *in vivo*, HCT116-SOX9(+), HCT116-SOX9(+)/S100P(−), and HCT116-oNC cells were injected subcutaneously into nude mice. As showed in Figure 5A, xenografted HCT116-SOX9(+) cells rapidly proliferated in mice compared with HCT116-oNC cells (*P* = 0.012). However, xenografted tumor growth was suppressed in mice injected with HCT116-SOX9(+)/S100P(−) cells compared to HCT116-SOX9(+) cells (*P* = 0.005, Figure 5A). HE and IHC staining confirmed SOX9 and S100P expression in the xenografted tumors (Figure 5B). Moreover, HCT116-SOX9(+) cells formed more peritoneal metastases than HCT116-oNC cells after 1 month (*P* = 0.013). More importantly, knockdown of S100P dramatically reduced the number of metastatic foci in the abdominal cavity of mice (*P* = 0.003) (Figure 5C). Therefore, these data suggested that S100P plays a pivotal role in SOX9-promoted metastasis and invasion of colon cancer.

### SOX9 or S100P overexpression in primary colon cancer is associated with adverse prognosis

**Figure 6:**
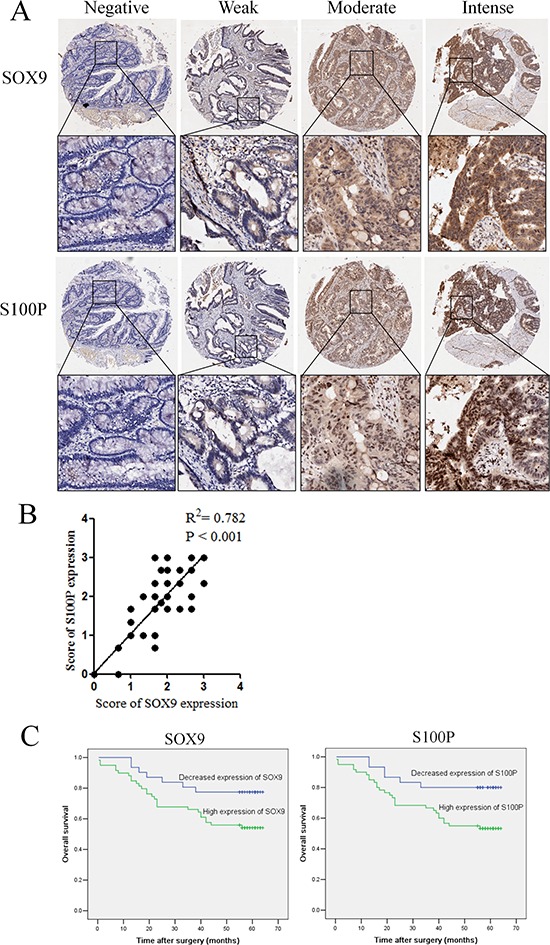
The coordinate expression of SOX9 and S100P in primary human colon cancers **A.** TMA analysis of SOX9 and S100P in primary human colon cancer tissues and normal adjacent tissues at ×100 and ×200 magnification. **B.** Regression analysis of SOX9 and S100P expression in primary colon cancers (*n* = 90). Linear regression analysis was performed based on the expression score of SOX9 and S100P from the TMA. **C.** Kaplan–Meier survival analysis of SOX9 and S100P expression in patients with colon cancer (log-rank test).

To explore the clinical relevance of SOX9 regulation of S100P expression in colon cancer, we performed IHC analysis of the tissue microarray (TMA) containing 90 pathologically annotated cases of colon cancer. The results showed that the expression of SOX9 and S100P in colon cancer had a high concordance (Figure 6A). After calculating the regression coefficient between the expression scores of SOX9 and S100P, we found that there was a significant linear regression between SOX9 and S100P (R^2^ = 0.782, *P* < 0.001) in primary colon cancer (Figure 6B). Based on the Kaplan-Meier Log-rank test, up-regulated expression of SOX9 (*P* = 0.037) or S100P (*P* = 0.020) was correlated with poor survival (Figure 6C). Univariate Cox regression model analysis revealed that poor survival was significantly associated with tumor size (hazard ratio, HR: 1.99, 95% confidence interval, 95%CI: 1.01-3.92; *P* = 0.047), differentiation (HR: 3.09, 95%CI: 1.43-6.65; *P* = 0.004), colonic wall invasion (HR: 2.46, 95%CI: 1.25-4.83; *P* < 0.009), nodal metastasis (HR: 3.20, 95%CI: 1.62-6.31; *P* = 0.001), AJCC (American Joint Commitee on Cancer) stage (HR: 3.87, 95%CI: 1.93-7.76; *P* < 0.001), S100P expression (HR: 2.72, 95%CI: 1.13-6.58; *P* = 0.026), and SOX9 expression (HR: 2.34, 95%CI: 1.02-5.38; *P* = 0.045). Based on the results of the univariate survival analysis, multivariate survival analysis was performed. After adjustment, tumor size, tumor differentiation, AJCC stage, SOX9 expression, and S100P expression were identified as covariates. Colonic wall invasion and nodal metastasis were excluded from the multivariate survival analysis due to their interactions with AJCC stage. AJCC stage was considered to be an independent risk predictor (HR: 3.37, 95%CI: 1.58-7.17; *P* = 0.002) for poor overall survival but not for SOX9 or S100P expression (Table 1). The clinical sample results expanded our findings in model systems showing that SOX9 and S100P are co-expressed and associated with metastasis and invasion, thereby influencing prognosis in colon cancer.

**Table 1:** Univariate and multivariate analyses of different prognostic factors in 90 patients with colon cancer in TMA

Variable	All case	Univariate analysis^1^		Multivariate analysis^1^	
		HR (95% CI)	*P*-value	HR (95% CI)	*P*-value
**Gender**		0.75 (0.38−1.49)	0.417		
Male	47				
Female	43				
**Age at diagnosis (years)**		0.79 (0.40−1.54)	0.487		
≦65	41				
>65	49				
**Size (diameter)**^2^**, cm**		1.99 (1.01−3.92)	0.047	2.22 (1.11−4.43)	0.024
≦5	49				
>5	40				
**Differentiation**		3.09 (1.43−6.65)	0.004	1.38 (0.60−3.17)	0.446
Well/moderate	76				
Poor	14				
**Colon wall invasion**		2.46 (1.25−4.83)	0.009		
Without breakthrough Serosa	63				
Serosa or Adjacent organs	27				
**Nodal metastasis**		3.20 (1.62−6.31)	0.001		
N0	59				
N1/N2	31				
**AJCC stage**		3.87 (1.93−7.76)	<0.001	3.37 (1.58−7.17)	0.002
I/II	57				
III/IV	33				
**S100P expression**		2.72 (1.13−6.58)	0.026	2.92 (0.83−10.26)	0.095
Decreased expression	30				
High expression	60				
**SOX9 expression**		2.34 (1.02−5.38)	0.045	0.976 (0.30−3.145)	0.968
Decreased expression	31				
High expression	59				

HR, hazard ratio; CI, confidence interval.

1 Cox regression model (method = Enter).

2 1 cases missing tumor size value.

## DISCUSSION

This study contributes to our understanding of the molecular mechanism by which S100P overexpression in colon cancer promotes tumor progression. Overexpression of S100P has been detected in several types of cancers [8–17]. To understand the regulation of S100P overexpression, we predicted and confirmed that SOX9 is a transcription factor for S100P. As a classical transcriptional factor, SOX9 may directly or indirectly regulate the transcription of target genes. Among many candidate genes we screened, S100P expression was selectively up-regulated by SOX9 in both colon cancer cell lines and human colon cancer samples. To demonstrate that S100P is induced as a result of SOX9 overexpression in colon cancer, we performed EMSA and quantitative ChIP assays, and we found that SOX9 protein binds to the potential binding site in the *S100P* promoter (AAACAAAAG). The direct regulation of *S100P* transcription by SOX9 was corroborated by a dual-luciferase reporter assay. Mutation analysis of the promoter-binding site indicated that overexpression of SOX9 activated *S100P* promoter activity. However, it remains unknown whether the binding specificity is determined by the adjacent sequences or other unknown cofactors.

To explore the biological function of SOX9 in colon cancer, we knocked down SOX9 expression in HCT116 cells via lentiviral-mediated transfection. The invasion and migration capacities of HCT116 cells were significantly decreased as a result of the down-regulation of SOX9 expression. In addition, low SOX9 expression was associated with decreased tumor growth and metastasis in the animal model. Western blot analysis showed that SOX9 knockdown was accompanied by down-regulation of S100P, down-regulation of RAGE, decreased phosphorylation of ERK1/2, and inhibition of the EMT process. S100P promotion of cancer progression via its specific role in cell proliferation, survival, angiogenesis, and metastasis in human cancers has been reported [18–21]. Signal transduction pathways and regulatory molecules mediating these effects include Ca2+ ions [22], RAGE-dependent pathways [23–25], ezrin [26], calcyclin-binding protein/Siah-1-interacting protein (CacyBP/SIP) [27, 28], and cathepsin D [29, 30]. S100P has been observed in the nucleus and/or the cytoplasm of various cell types [21, 31]. Extracellular S100P activates RAGE, which is a multiligand transmembrane receptor of the immunoglobulin super family, and it has been identified as a factor that influences the characteristics of various cancer cell types [32–35]. Cromolyn has been reported to bind S100P, prevent activation of RAGE, inhibit tumor growth, and increase the effectiveness of gemcitabine in pancreatic cancer experimental models [36, 37]. Previous research has suggested that the phosphorylation level of ERK1/2 has a close relationship with SOX9 expression and the EMT process [38–40]. Therefore, we concluded that SOX9 induces the expression of S100P, which consequently activates its direct target, RAGE, and phosphorylates ERK1/2, subsequently leading to EMT, which promotes tumor invasion and metastasis.

Our study showed that SOX9 regulation of S100P promotes cancer cell migration and invasion as well as inducing EMT. However, it was unclear if S100P is necessary for the functions of SOX9 in cancer progression. By constructing a SOX9-overexpressing and S100P knockdown colon cancer cell line, we confirmed that knockdown of S100P expression can dramatically decrease SOX9-mediated migration and invasion abilities. An *in vivo* assay demonstrated that SOX9-promoted peritoneal metastasis was blocked by S100P knockdown, indicating that up-regulation of S100P expression at least partially accounts for the function of SOX9 in promotion of tumor progression *in vivo*. Moreover, the linear regression between SOX9 and S100P expression was pronounced in colon cancer TMA analysis. Further survival analysis indicated that overexpression of SOX9 or S100P predicted poor prognosis. Although overexpression of SOX9 or S100P could be a survival risk factor in the Cox univariate analysis, the prognosis predictive power of SOX9 or S100P expression could not exceed the classical AJCC stage in the Cox multivariate analysis. However, the clinical utility of SOX9 or S100P as a prognostic biomarker compared with conventional factors remains to be verified in prospective studies analyzing many more cases.

In conclusion, we demonstrated that SOX9 and S100P are both overexpressed in colon cancer. SOX9, as a transcription factor of S100P, up-regulates the transcription activity of S100P and promotes metastasis, invasion and poor survival of colon cancer by regulating S100P expression. More importantly, S100P plays a pivotal role in SOX9-induced metastasis and invasion of colon cancer.

## MATERIALS AND METHODS

### Tissue samples and cell culture

Human colon cancer tissue samples were obtained from the Department of General Surgery at Nanfang Hospital (Guangzhou, China) with approval by the Institute Research Medical Ethics Committee of Nanfang Hospital. Tissue samples were collected from patients who were diagnosed with colon adenocarcinoma. Adjacent normal tissues that were at least 5 cm away from the tumor margins and confirmed to be free of tumor deposits were used as normal control in this study. HCT 116 cells were obtained from the Cell Bank of Type Culture Collection (Shanghai City, China) and were cultured in DMEM (HyClone) supplemented with 10% fetal bovine serum (FBS; Gibco), 100 U/mL penicillin, and 100 μg/mL streptomycin at 37°C in a humidified 5% CO_2_ incubator.

### Western blot analysis and real-time Q-PCR

Proteins were separated on SDS-PAGE gels followed by transfer to polyvinylidene fluoride membranes. The following antibodies were used for Western blotting: monoclonal rabbit S100P antibody and polyclonal rabbit anti-SOX9 obtained from Abcam; and RAGE, p-ERK1/2, ERK1/2, E-cadherin, N-cadherin, Vimentin, Snail, and GAPDH antibodies obtained from Cell Signaling Technology. Blots were incubated with the appropriate primary antibody in TBS-Tween 20 at 4°C overnight. Following incubation with an anti-mouse or anti-rabbit secondary antibody conjugated to horseradish peroxidase, the blots were visualized using the Luminata Chemiluminescent Detection Kit (Millipore). cDNA was synthesized from 1 μg of total RNA for reverse transcription using a PrimeScript RT Master Mix Perfect Real Time Kit (Takara). Oligo (dT) primers were synthesized by Takara (Supplementary Table S1). Q-PCR was performed to assess gene expression using a SYBR Premix Ex Taq II Kit (Takara).

### Verification of the binding between SOX9 and *S100P* gene promoter

Immunofluorescence, electrophoretic mobility shift assay (EMSA), chromatin-immunoprecipitation (ChIP) assay, and dual-luciferase reporter assay were performed to test if SOX9 can bind and transcriptionally activate the S100P gene promoter. Biotin-labeled, unlabeled, and mutant probes were designed for EMSA. A SOX9-overexpressing plasmid and luciferase reporter plasmids containing wild type (S100P-promoter-WT) or mutant (S100P-promoter-MU) S100P promoter sequences were constructed for the dual-luciferase reporter assay. All of the experiments followed standard protocols. See supplementary methods for more details.

### Establishment of stable cell lines

An optimized SOX9 knockdown lentivirus expressing SOX9-siR-2167 (Supplementary Table S1, Supplemental Figure S1) and the green fluorescent protein (EGFP) gene (GeneChem) was used to stably transfect the HCT116 cell line (HCT116-SOX9(−)). Cells were transfected with negative control lentivirus (GeneChem) as a negative control (HCT116-iNC). The SOX9-overexpressing cell line (HCT116-SOX9(+)) was constructed using the lentivirus expressing SOX9 cDNA (GeneChem), and a negative control (HCT116-oNC) cell line was also generated. To establish the SOX9-overexpressing and S100P knockdown stable cell line (HCT116-SOX9(+)/S100P(−)), lentiviruses expressing SOX9 cDNA and S100P-siR-250 (Supplementary Table S1) were co-transfected into the HCT116 cell line. All of the lentiviruses were then mixed with 5 μg/mL polybrene to enhance the transfection efficiency. After 72 h, Western blotting was performed to detect the expression of the *SOX9* and *S100P* genes.

### Cell migration and invasion assays

For migration assays, cell culture inserts equipped with 8-μm membranes were used (BD Biosciences). For invasion assays, BioCoat Matrigel invasion chambers (BD Biosciences) were used according to the manufacturer's protocol. Cells (5 × 10^5^) were seeded into the cell culture inserts in serum-free medium, and the lower chamber was filled with medium containing 15% FBS. The cells were allowed to migrate for 48 h at 37°C. After removing the cells that remained in the top chamber, the top surface of each membrane was cleared of cells with a cotton swab. Cells that had penetrated to the bottom side of the membrane were then fixed in paraformaldehyde, stained with crystal violet, and counted in nine randomly selected microscope fields (×200) per well.

### Animal models

To evaluate *in vivo* tumor tumorigenesis, 5 × 10^6^ cells in 0.2 mL of serum-free DMEM were injected subcutaneously into the left flank or right flank of nude mice (three mice in each group). Tumors were measured with calipers from day 5 to day 31 after injection, and the tumor volumes were calculated according to the following formula: 0.5 × length × width^2^ [9]. For the metastasis assay, nude mice were injected intraperitoneally with 1 × 10^7^ cells in 0.2 mL of serum-free DMEM (three mice in each group). One month later, the mice were sacrificed, and intraperitoneal tumor formation was assessed using the In-Vivo F Imaging System (Kodak). All animal experiments were undertaken in accordance with the National Institutes of Health Guide for the Care and Use of Laboratory Animals with the approval of the Laboratory Animal Centre of the Southern Medical University in Guangzhou, China. All animals were housed in a virus-free facility and maintained in a standard temperature- and light-controlled animal facility.

### Tissue microarray and immunohistochemistry

The TMA containing a total of 90 colon cancer patients, together with the data of pathological staging in accordance with TNM classification of the American Joint Committee on Cancer (AJCC, 2010) and survival time after surgery for all cases (Supplemental Table S3), was obtained from the National Engineering Center For Biochip at Shanghai.

The standard two-step immunohistochemistry (IHC) technique was used in the present study. Sections of the TMA were subjected to IHC with anti-S100P (Abcam) and anti-SOX9 (Abcam) antibodies. SOX9 and S100P expression was analyzed independently by three observers (Z. S., H. D., and Y. F.) using the same light microscope (OLYMPUS BX51). Staining was evaluated according to the criteria described by previous studies [41–43]. The SOX9 and S100P staining results were classified according to the carcinoma cell staining intensity as follows: 0, negative staining; 1, weak staining; 2, moderate staining; and 3, intense staining. We defined negative- and weak-stained cells as low expressers, and cells that were moderately and intensely stained were considered to be high expressers of this protein. The average score for each sample evaluated by three observers was considered as the final IHC score.

### Statistical analysis

Statistical analysis was performed using the SPSS statistical software package (standard version 13.0; SPSS, Chicago, IL). T-tests were used to evaluate differences between established stable cell lines. Linear regression analysis was performed to assess the relationship between SOX9 and S100P in the TMA. Survival curves were obtained using the Kaplan–Meier method. Cox proportional hazards regression was performed to identify independent factors with a significant impact on patient survival. Probability values from the two-tailed test less than 0.05 were considered significant.

## SUPPLEMENTARY DATA




