# Supplementary material for: Identification of the interplay between SOX9 and S100P in the metastasis and invasion of colon carcinoma
Source: Oncotarget. 2015 May 13;6(24):20672–84. doi: 10.18632/oncotarget.3967 (PMC4653034; doi:10.18632/oncotarget.3967)
Supplement: Supplementary file 1 [file oncotarget-06-20672-s001.pdf]

SUPPLEMENTARY DATA

Immunofluorescence

Immunofluorescence was used to determine the localization of SOX9 in colon cancer cells. Cells were seeded at 5 × 10^4 cells per 13-mm coverslip in a 24-well plate and cultured overnight. The cells were then fixed in 4% paraformaldehyde and permeabilized with 0.1% Triton X-100. After blocking in 1% bovine serum albumin, the cells were incubated with mouse anti-SOX9 (Abcam). The secondary antibodies included anti-mouse IgG (H+L) F(ab')2 fragment (Alexa Fluor 488 Conjugate) and anti-rabbit IgG (H+L) F(ab')2 fragment (Alexa Fluor® 555 Conjugate; Cell Signaling Technology). DNA was stained with 1 µg/mL 4′,6-diamidino-2-phenylindole (DAPI; Cell Signaling Technology). A series of optical sections were acquired and projected to single images using an Olympus FV10i-W.

Electrophoretic mobility shift assay (EMSA)

Nuclear proteins from the HCT116 cell line were extracted using a nucleoprotein extraction kit (BestBio). The sequences of the *S100P* gene probes were designed based on the predicted transcription factor-binding site (TFBS) of SOX9 for the *S100P* gene. EMSAs were performed according to standard protocols and visualized using a Chemiluminescent EMSA Kit (Beyotime Biotechnology). The specificity of binding was examined by competition with 100-fold excess of unlabeled probe and mutant probe. For supershift assays, the nuclear extract was incubated at room temperature for 20 min with a biotin-labeled probe and antibodies against SOX9 (Abcam). Probe/nuclear protein complexes were separated by electrophoresis on a non-denaturing PAGE gel, and the resolved products were transferred onto a positively charged nylon membrane (Millipore). Bands were detected with the Luminata Chemiluminescent Detection Kit (Millipore).

Chromatin immunoprecipitation (ChIP) assay

Quantitative chromatin immunoprecipitation (Q-ChIP) was performed following the protocol from an EZ-ChIP Chromatin Immunoprecipitation Kit (Millipore). The protein/DNA complex was isolated from the HCT116 cell line and sheared by sonication to lengths of 200 to 1, 000 base pairs. Anti-SOX9 (Abcam) was used to precipitate the complex, and normal IgG was used as the negative control. The DNA-protein crosslinks were reversed by heating at 65°C for 4 h. After phenol and chloroform extraction, DNA was purified by ethanol precipitation. Real-time PCR analysis was performed using the selected primers.

Dual-luciferase reporter assay

To generate luciferase reporter plasmids containing the S100P promoter sequences, DNA fragments containing wild type (S100P-promoter-WT) or mutant (S100P-promoter-MU) S100P promoter sequence (nucleotide from –1500 to +55) were amplified using genomic DNA. The PCR products were cloned into the pGL3-promoter vector by Genechem (Shanghai, China). The mutant sequence of the S100P promoter replaced the predicted TFBS of "AAACAAAAG" to "ACGCAGCAT". To construct SOX9-overexpressing plasmids, human SOX9 cDNA was inserted into pMSCV-GFP-IRES. The activity of the reporter was measured using a Dual-Luciferase™ reporter system (Promega). HCT-116 cancer cells were seeded at a density of 2 × 10^4 cells in a 24-well plate. Cells were cotransfected with 0, 0.2 or 0.4 µg of SOX9-overexpressing vector and 0.2 µg of S100P-promoter-WT or S100P-promoter-MU. The transfection was performed using X-tremegene HP (Roche) according to the manufacturer's protocol. After incubation for 24 h, the cells were lysed. Luciferase activity was determined by the Dual-Luciferase Reporter Assay System as described in the manufacturer's instructions (Promega). *Renilla* luciferase was used to normalize transfection efficiency.

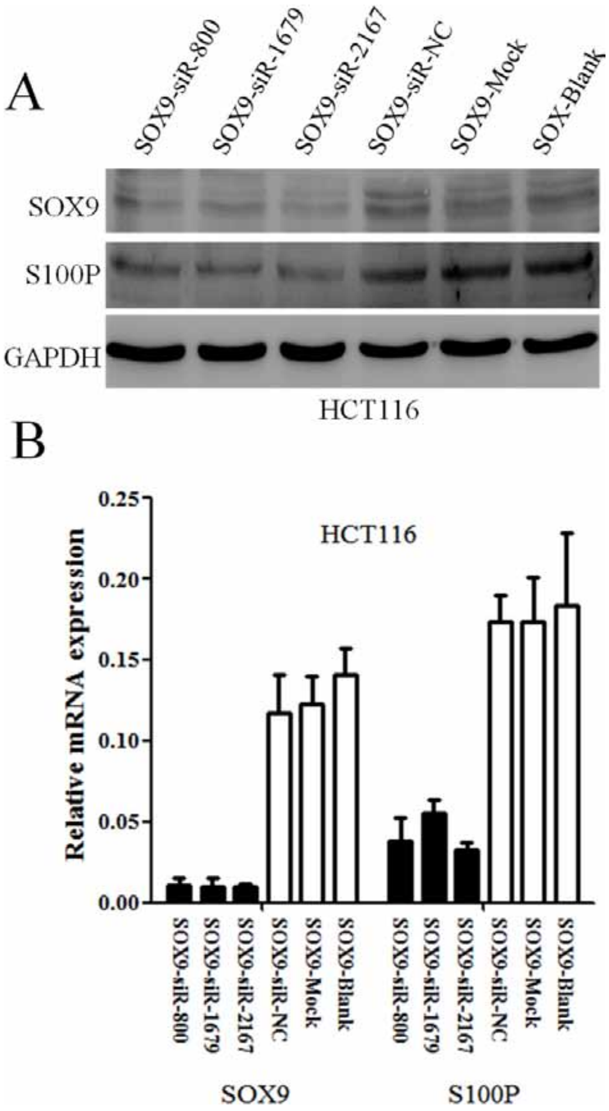

Supplementary Figure S1: Interference of SOX9 expression results in down-regulation of S100P expression in the HCT116 cell line. A. After SOX9 was transiently knocked down by three different SOX9-targeted siRNAs, the expression levels of both SOX9 and S100P were down-regulated in HCT116 cells as detected by Western blot analysis. **B.** SOX9 and S100P mRNA expression levels were also decreased after SOX9 was knocked down by siRNAs as detected by Q-PCR.

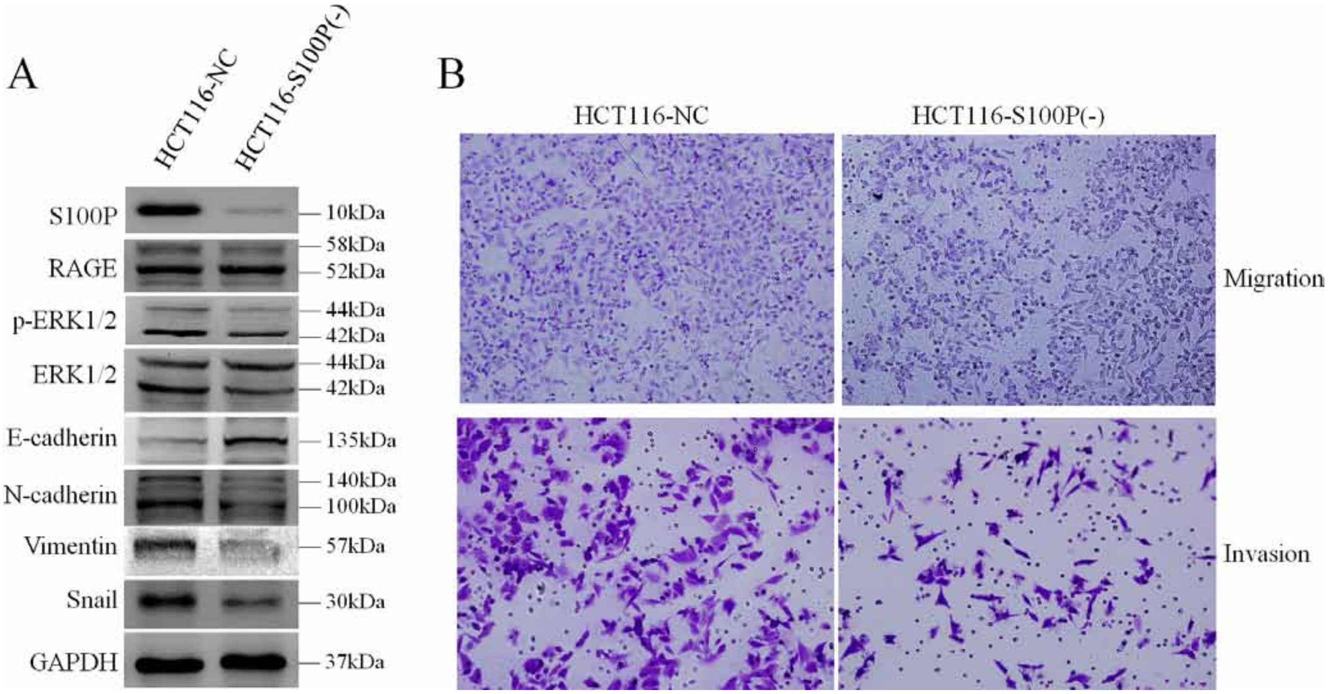

Supplementary Figure S2: Effects of S100P knockdown on EMT, invasion and metastasis. A. Markers of EMT were detected using Western blot analysis in the HCT116-S100P(-) cell line (cell line with stable lentiviral suppression of S100P expression). **B.** The invasion and metastasis of HCT116-S100P(-) cells compared with negative control cells (HCT116-NC) were measured by Transwell and Borden chamber assays.

Supplementary Table S1. Primer sequences used for the ChIP assay

Oligo (dT) primers for Q-PCR in Q-ChIP	
Forward primer	5′-TCTGGGTTTTATGGCAGGGTTT-3′
Reverse primer	5′-GAGCCTTTGTGCCGTCTAAGC-3′

Supplementary Table S2. The sequence of Oligo(dT) primers for Q-PCR and siRNAs for SOX9 and S100P interference

**Oligo (dT) primers for Q-PCR**	
S100P- Forward	5′-TGCAGAGTGGAAAAGACAAGGAT-3′
S100P- Reverse	5′-CCACCTGGGCATCTCCATT-3′
SOX9- Forward	5′-GGAGATGAAATCTGTTCTGGGAATG-3′
SOX9- Reverse	5′-TTGAAGGTTAACTGCTGGTGTTCTG-3′
Oligo (dT) primers for Q-PCR in Q-ChIP	
Forward primer	5′-TCTGGGTTTTATGGCAGGGTTT-3′
Reverse primer	5′-GAGCCTTTGTGCCGTCTAAGC-3′
siRNAs for SOX9 and S100P for constructing the lentivirus	
SOX9-siR-2167	5′-GCGAAAUCAACGAGAAACUTT-3′<br>5′-AGUUUCUCGUUGAUUUCGCTT-3′
S100P-siR-250	5′-CCGUGGAUAAAUUGCUCAATT-3′<br>5′-UUGAGCAAUUUAUCCACGGTT-3′

Supplementary Table S3. The clinicopathological characteristics of clinical samples in TMA
