# Supplementary material for: Identification of the interplay between SOX9 and S100P in the metastasis and invasion of colon carcinoma
Source: Oncotarget. 2015 May 13;6(24):20672–84. doi: 10.18632/oncotarget.3967 (PMC4653034; doi:10.18632/oncotarget.3967)
Supplement: Supplementary file 2 [file oncotarget-06-20672-s002.doc]

Table S3. The clinicopathological characteristics of clinical samples in TMA

| Sample ID | Status | Survival time (months) | Gender | Age (years) | TNM stage | Differentiation | Tumor size (cm) | AJCC stage | SOX9 score | S100P score |
| --- | --- | --- | --- | --- | --- | --- | --- | --- | --- | --- |
| 1454 | Alive | 64 | Male | 72 | T3N0M0 | Moderate | 7 | IIA | 1.83 | 1.83 |
| 1455 | Dead | 1 | Female | 57 | T2N1M0 | Moderate | 7 | IIIA | 2.33 | 2.33 |
| 1456 | Dead | 13 | Male | 76 | T3N0M0 | Moderate | 6 | IIA | 2.00 | 3.00 |
| 1458 | Alive | 64 | Female | 63 | T2N0M0 | Moderate | 5 | IIA | 2.00 | 2.00 |
| 1461 | Alive | 63 | Female | 78 | T3N3M0 | Moderate | 7 | IIIC | 3.00 | 3.00 |
| 1462 | Dead | 22 | Male | 78 | T4N0M0 | Moderate | 8 | IV | 2.00 | 2.00 |
| 1464 | Dead | 7 | Male | 63 | T3N3M0 | Poor | 6 | IIIC | 2.00 | 2.00 |
| 1502 | Alive | 63 | Female | 68 | T3N0M0 | Moderate | 6 | IIA | 0.67 | 0.67 |
| 1503 | Alive | 63 | Male | 39 | T3N0M0 | Moderate | 6 | IIB | 2.33 | 2.67 |
| 1504 | Dead | 23 | Male | 68 | T2N0M0 | Moderate | 6 | I | 3.00 | 3.00 |
| 1505 | Alive | 63 | Male | 62 | T2N0M0 | Moderate | 3 | IIA | 2.00 | 2.00 |
| 1508 | Alive | 63 | Male | 78 | T3N0M0 | Moderate | 5 | IIA | 2.67 | 2.67 |
| 1510 | Dead | 44 | Female | 50 | T3N0M0 | Moderate | 4 | IIB | 2.00 | 2.00 |
| 1556 | Alive | 62 | Male | 73 | T3N0M0 | Moderate | 11 | IIA | 1.67 | 1.00 |
| 1557 | Dead | 38 | Male | 68 | T3N0M0 | Moderate | 6 | IIB | 2.33 | 1.67 |
| 1558 | Dead | 13 | Female | 87 | T3N1M | Moderate | 6 | IIIC | 2.00 | 2.67 |
| 1559 | Dead | 8 | Female | 52 | T3N0M0 | Poor | 7 | IIB | 2.67 | 3.00 |
| 1560 | Alive | 62 | Female | 51 | T1N0M0 | Moderate | 3 | I | 2.00 | 2.00 |
| 1561 | Dead | 56 | Male | 55 | T3N2M0 | Moderate | 4 | IIIC | 2.00 | 2.33 |
| 1562 | Dead | 17 | Male | 73 | T4N0M0 | Poor | 7 | IV | 2.67 | 3.00 |
| 1563 | Alive | 62 | Female | 61 | T3N0M0 | Moderate | 3 | IIA | 2.67 | 2.00 |
| 1564 | Dead | 12 | Female | 48 | T3N0M0 | Poor | 2 | IIA | 2.67 | 3.00 |
| 1565 | Alive | 62 | Female | 59 | T2N0M0 | Moderate | 3 | I | 0.67 | 0.00 |
| 1566 | Dead | 40 | Female | 77 | T2N0M0 | Moderate | 4 | I | 2.00 | 2.00 |
| 1567 | Dead | 42 | Male | 78 | T3N1M0 | Moderate | 5 | IIIB | 2.67 | 3.00 |
| 1570 | Alive | 62 | Male | 31 | T3N1M0 | Poor | 4 | IIIB | 0.00 | 0.00 |
| 1571 | Dead | 33 | Female | 79 | T3N0M0 | Moderate | 7 | IIA | 1.67 | 1.00 |
| 1572 | Alive | 61 | Male | 81 | T3N1M0 | Moderate | 4 | IIIB | 1.00 | 1.00 |
| 1573 | Alive | 61 | Male | 85 | T3N0M0 | Moderate | 4 | IIA | 1.00 | 1.00 |
| 1574 | Dead | 40 | Male | 90 | T3N0M0 | Moderate | 7 | IIB | 2.67 | 3.00 |
| 1576 | Alive | 61 | Female | 70 | T2N0M0 | Moderate | 5 | I | 2.67 | 2.67 |
| 1577 | Dead | 23 | Female | 66 | T4N1M0 | Poor | 5 | IIIC | 2.67 | 3.00 |
| 1579 | Alive | 61 | Male | 73 | T2N0M0 | Moderate | 4 | IIA | 3.00 | 3.00 |
| 1614 | Alive | 61 | Female | 54 | T2N0M0 | Moderate | 4 | IIA | 2.00 | 3.00 |
| 1615 | Alive | 61 | Male | 50 | T1N0M0 | Well | 4 | I | 2.00 | 1.67 |
| 1616 | Alive | 61 | Female | 74 | T2N0M0 | Well | 5 | IIA | 3.00 | 2.33 |
| 1617 | Alive | 61 | Male | 80 | T3N0M0 | Moderate | 8 | IIA | 1.00 | 1.00 |
| 1619 | Alive | 61 | Female | 65 | T2N0M0 | Moderate | 4 | IIA | 1.00 | 1.67 |
| 1620 | Alive | 61 | Female | 59 | T2N0M0 | Moderate | 5 | IIA | 2.00 | 2.00 |
| 1622 | Alive | 61 | Male | 79 | T2N0M0 | Moderate | 4 | IIA | 2.00 | 1.67 |
| 1624 | Dead | 13 | Female | 76 | T3N1M0 | Moderate | 4 | IIIB | 1.00 | 1.00 |
| 1625 | Alive | 60 | Female | 76 | T3N0M0 | Moderate | 8 | IIA | 3.00 | 3.00 |
| 1626 | Dead | 39 | Male | 63 | T3N1M0 | Moderate | 5 | IIIB | 3.00 | 3.00 |
| 1628 | Dead | 25 | Female | 76 | T3N1M0 | Moderate | 8 | IIIB | 1.00 | 1.00 |
| 1629 | Alive | 61 | Male | 56 | T2N0M0 | Moderate | 4 | IIA | 1.67 | 0.67 |
| 1630 | Alive | 60 | Female | 44 | T2N0M0 | Moderate | 8 | I | 2.67 | 3.00 |
| 1663 | Dead | 13 | Male | 73 | T3N0M0 | Moderate | 6 | IIA | 1.00 | 1.33 |
| 1668 | Alive | 60 | Female | 66 | T1N1M0 | Moderate | 8 | IIIA | 1.33 | 1.00 |
| 1669 | Dead | 1 | Female | 48 | T3N0M0 | Moderate | 8 | IIA | 2.00 | 2.00 |
| 1673 | Alive | 59 | Male | 68 | T2N0M0 | Moderate | 4 | IIA | 1.00 | 1.00 |
| 1732 | Alive | 59 | Male | 79 | T3N0M0 | Moderate | 6 | IIA | 0.00 | 0.00 |
| 1733 | Alive | 59 | Male | 55 | T3N1M0 | Poor | 5 | IIIB | 3.00 | 3.00 |
| 1735 | Alive | 59 | Female | 65 | T3N0M0 | Moderate | 4 | IIA | 1.67 | 1.67 |
| 1740 | Dead | 7 | Female | 73 | T3N1M0 | Moderate | 8 | IIIB | 2.00 | 2.00 |
| 1741 | Alive | 59 | Male | 81 | T3N1M0 | Moderate | 8 | IIIB | 1.83 | 2.67 |
| 1742 | Alive | 59 | Male | 61 | T2N0M0 | Moderate | 5 | I | 2.00 | 1.67 |
| 1743 | Dead | 16 | Male | 65 | T4N1M0 | Poor | 6 | IIIC | 2.67 | 1.67 |
| 1744 | Dead | 16 | Male | 61 | T3N2M1 | Poor | 4 | IV | 3.00 | 3.00 |
| 1745 | Alive | 59 | Male | 80 | T3N0M0 | Moderate | 4 | IIA | 2.67 | 3.00 |
| 1756 | Alive | 58 | Female | 71 | T3N1M0 | Moderate | 3 | IIIB | 3.00 | 3.00 |
| 1758 | Alive | 58 | Female | 55 | T3N0M0 | Poor | 11 | IIA | 2.33 | 2.33 |
| 1762 | Alive | 58 | Female | 69 | T3N0M0 | Moderate | 8 | IIB | 2.67 | 3.00 |
| 1764 | Alive | 58 | Female | 80 | T3N1M0 | Poor | 6 | IIIB | 2.67 | 3.00 |
| 1765 | Dead | 21 | Male | 55 | T3N0M0 | Moderate | 4 | IIB | 3.00 | 3.00 |
| 1767 | Alive | 58 | Male | 83 | T3N0M0 | Well | 5 | IIA | 2.00 | 1.67 |
| 1811 | Alive | 57 | Male | 73 | T3N0M0 | Well | 7 | IIB | 1.67 | 2.33 |
| 1813 | Alive | 57 | Female | 82 | T3N1M0 | Poor | 4 | IIIB | 3.00 | 3.00 |
| 1814 | Alive | 57 | Female | 69 | T2N0M0 | Moderate | 2 | I | 1.00 | 1.00 |
| 1815 | Alive | 57 | Female | 46 | T3N0M0 | Moderate | 6 | IIA | 2.67 | 3.00 |
| 1819 | Alive | 57 | Female | 56 | T3N0M0 | Moderate | 5 | IIA | 2.67 | 3.00 |
| 1820 | Alive | 56 | Female | 78 | T3N0M0 | Moderate | 5 | IIA | 2.00 | 2.00 |
| 1836 | Alive | 56 | Female | 81 | T3N0M0 | Moderate | 6 | IIB | 1.33 | 1.00 |
| 1839 | Alive | 56 | Male | 73 | T3N0M0 | Moderate | 5 | IIB | 3.00 | 3.00 |
| 1841 | Alive | 56 | Female | 50 | T3N0M0 | Moderate | 6 | IIA | 1.33 | 2.00 |
| 1904 | Dead | 19 | Female | 27 | T3N2M0 | Poor | 4 | IIIC | 1.67 | 3.00 |
| 1907 | Dead | 35 | Male | 54 | T3N0M0 | Moderate | 5 | IIA | 2.67 | 2.67 |
| 1914 | Alive | 55 | Female | 77 | T3N1M0 | Moderate | NA | IIIB | 1.67 | 2.00 |
| 1915 | Alive | 55 | Female | 55 | T3N1M0 | Moderate | 9 | IIIB | 1.67 | 1.67 |
| 1917 | Alive | 55 | Male | 66 | T2N0M0 | Moderate | 3 | I | 1.67 | 1.67 |
| 1918 | Dead | 42 | Male | 60 | T3N3M0 | Moderate | 4 | IIIC | 2.67 | 2.67 |
| 1919 | Dead | 19 | Male | 65 | T3N2M0 | Moderate | 5 | IIIB | 2.67 | 3.00 |
| 1921 | Dead | 15 | Male | 56 | T3N1M0 | Moderate | 6 | IIIB | 2.67 | 2.67 |
| 1923 | Alive | 55 | Male | 54 | T3N1M0 | Moderate | 7 | IIIB | 2.00 | 2.00 |
| 1927 | Dead | 19 | Male | 67 | T3N1M0 | Moderate | 6 | IIIB | 1.00 | 1.00 |
| 1928 | Alive | 55 | Female | 52 | T2N0M0 | Moderate | 6 | I | 1.00 | 1.00 |
| 1929 | Dead | 23 | Female | 62 | T3N1M0 | Moderate | 5 | IIIB | 2.00 | 2.00 |
| 1990 | Alive | 57 | Male | 43 | T3N0M0 | Moderate | 4 | IIA | 2.00 | 2.00 |
| 1991 | Alive | 57 | Male | 83 | T3N0M0 | Moderate | 4 | IIB | 2.67 | 2.67 |
| 1992 | Alive | 56 | Male | 66 | T3N0M0 | Moderate | 4 | IIA | 1.67 | 1.67 |
| 1993 | Dead | 0 | Male | 82 | T3N2M0 | Poor | 7 | IIIC | 2.00 | 2.33 |
